# Barth Syndrome: Psychosocial Impact and Quality of Life Assessment

**DOI:** 10.3390/jcdd9120448

**Published:** 2022-12-09

**Authors:** Anandbir Bath, Oguz Akbilgic, David Wilbanks, Jay Patel, Morgan Wallen, Shereen Haji, Arnab Das, John Alexander, Issa Pour-Ghaz, Deya Alkhatib, Yonglin Huang, Erik Lontok, John Jefferies

**Affiliations:** 1 Department of Cardiovascular Medicine, University of Tennessee Health Science Center, Memphis, TN 38163, USA; 2 Section of Cardiovascular Medicine, Department of Internal Medicine, Wake Forest School of Medicine, Winston-Salem, NC 27157, USA; 3 Clinical Research Coordinator, Research Department, Barth Syndrome Foundation, Larchmont, NY 10538, USA; 4 Director of Research, Research Department, Barth Syndrome Foundation, Larchmont, NY 10538, USA

**Keywords:** Barth syndrome, cardiomyopathy, quality of life, anxiety, depression, genetics, psychosocial

## Abstract

Background: Barth syndrome (BTHS) is a rare X-linked genetic disease that affects multiple systems and leads to complex clinical manifestations. Although a considerable amount of research has focused on the physical aspects of the disease, less has focused on the psychosocial impact and quality of life (QoL) in BTHS. Methods: The current study investigated caregiver- (*n* = 10) and self-reported (*n* = 16) psychological well-being and QoL in a cohort of BTHS-affected patients and families. Participants completed the depression and anxiety components of the Patient-Reported Outcomes Information System (PROMIS) Short Form 8A and Health-related quality of life (HRQoL) surveys at enrollment and again during a follow-up period ranging from 6 to 36 months after baseline. Results: Quality of life changed significantly over time and the various domains with some improvement and some decline. Among the available caregiver-patient dyad data, there was a trend toward discordance between caregiver and self-reported outcomes. Most notably, patients reported improvement in HRQoL, while caregivers reported declines. This suggests that there may be differences in perceived quality of life between the patients and parents, though our study is limited by small sample size. Conclusion: Our study provides valuable insights into the impacts of psychosocial and mental health aspects of BTHS. Implications of these findings include incorporating longitudinal assessment of QoL and screening for psychological symptoms in BTHS care to identify interventions that may drastically impact health status and the course of the disease.

## 1. Introduction

Barth Syndrome (BTHS) is a recessive X-linked disease characterized by neutropenia, skeletal myopathy, cardiomyopathy, growth retardation, and debilitating fatigue [1,2]. It was first detailed by Dr. Peter Barth in 1983 but potentially was described in a 1979 report of an infant with heart failure and cardiomyopathy who was found to have abnormal mitochondria in the liver, kidney, and skeletal muscle upon autopsy [3,4]. The 1979 report identified several male family members who died in infancy and had cardiac disease.

BTHS results from mutations in the *TAFAZZIN* gene of the distal portion of chromosome Xq28 [5]. *TAFAZZIN* encodes an enzyme involved in the formation of mitochondrial phospholipid cardiolipin (CL) [6]. CL is involved in mitochondrial structure, mitochondrial apoptosis, optimization of cellular energy production, formation and assembly of mitochondrial cristae, and stabilization of the electron transport chain [7,8,9,10,11,12]. CL has been found in highly oxidative tissue such as skeletal and cardiac muscle. Pathogenic variants in *TAFAZZIN* result in an increased level of an intermediate CL species called monolysocardiolipins (MLCL) and a reduced amount of CL. The resulting increase in the ratio of MLCL:CL is unique to BTHS and further serves as a specific and sensitive diagnostic test [13,14].

While considerable research has focused on specific aspects of BTHS, such as the management of neutropenia and heart failure, much less research has investigated its psychosocial impact. Aside from the physical burden of BTHS, the disease imposes immense psychosocial consequences on the patient and family. For example, one study in BTHS patients (*n* = 34, Median age = 12.2 years; Range = 2–25) found that this patient group had lower health-related quality of life scores (i.e., school function and physical health) compared to healthy controls [15]. Impairments in neurocognitive performance may start as early as kindergarten [16,17]. Another study found that BTHS patients (*n* = 27, Mean age = 18.1 years, Range = 9–32) exhibited worse functioning across physical, psychosocial, and social domains, as well as the total quality of life scores relative to diabetes and cancer patients [16]. It is well known that the presence of chronic conditions in adults and children is associated with higher rates of depression and anxiety that can impair daily functioning and can result in higher health care utilization [18,19,20]. Worsening of these mental health conditions can lead to worse outcomes with patients becoming noncompliant with medical recommendations [21]. Moreover, internalizing symptoms, which include depression and anxiety, have been linked to poorer quality of life in BTHS patients. Patients report feeling socially isolated, resulting in reduced motivation to pursue future activities and harmful actions to cope via self-medication or self-destructive behaviors [22]. This is potentially due to difficulty in performing physically strenuous social activities, experiencing bullying and other social obstacles, and experiencing academic challenges [23]. Given the paucity of research on the psychosocial functioning of individuals with BTHS, more research in this area with both patient and caregiver reports is warranted to elucidate the psychosocial impact of BTHS and further inform and improve clinical care.

Overall, these findings indicate the enormous psychosocial impacts of BTHS on the patient and family. It is well established that individuals with chronic illnesses are at higher risk of developing anxiety and depression. There is an interplay between chronic illnesses’ precipitation of anxiety and depression and exacerbation of these illnesses due to psychosocial matters [24]. Despite this evidence, little attention has been given to this aspect in BTHS patients.

In this study, we further investigated the psychosocial impact of BTHS by capturing patient- and caregiver-reported information on the quality of life and psychological well-being. Our aims were twofold: (1) to characterize the prevalence of anxiety, depression, and health-related quality of life disparities in individuals suffering from BTHS so this information can be utilized to enhance clinical decision-making as well as aid in understanding the target population, and (2) identify deficits in patient care and aim to address these issues through the implementation of a BTHS-specific symptom checklist. Based on prior literature, we predicted that scores across the quality of life and psychological well-being measures would be abnormal in this standalone cohort. To our knowledge, this would be the first study to assess QoL and psychological components of BTHS using multiple timepoints and respondents. Through these assessments, we hope to eliminate gaps in clinical care and provide a route to improving patient outcomes.

## 2. Materials and Methods

### 2.1. Participants

The investigators partnered with the Barth Syndrome Foundation for the recruitment of the participants. To qualify for the study, participants must either by an individual diagnosed with Barth syndrome or a caregiver of a Barth syndrome-affected individual. To date, the Barth Syndrome Foundation has identified less than 125 individuals living in the U.S. with BTHS. Interested participants and families provided consent and assent to participate in this research study and were then provided links to the study questionnaires and followed up thereon. A total of 10 caregiver and 16 patient participants (approximately 13% of all known living U.S. cases) were recruited for the study (Mean age = 21.25; SD = 12.66) (Table 1). The final cohort consisted of participants with completed questionnaires and at least one follow-up at a later time.

### 2.2. Health-Related Quality of Life

The data for our research was collected with the administration of health-related quality of life (HRQoL) questionnaires which aimed to gather patients’ and caregivers’ beliefs about HRQoL, anxiety, and depressed moods. To assess HRQoL, we used the Peds-QL questionnaire, which contains 4 subscales to assess function across physical, emotional, social and school domains [25]. The responses were rated on a 5-point Likert scale ranging from 0 (never a problem) to 4 (almost always a problem). Subscale scores are derived by reverse scoring, then linearly transforming responses to a 0 to 100 scale, and then averaged. A higher score indicates better HRQoL. The total score is derived from the linearly transforming average of the 23 scales.

### 2.3. Depression and Anxiety

For assessment of depression and anxiety, we used pediatric (Short Form 8A version 2.0), adult (Short Form 8A), and parent-proxy instruments developed by Patient-Reported Outcomes Measurement Information System (PROMIS) [26]. The responses were rated on a 5-point Likert scale ranging from 1 (Never) to 5 (Almost Always) and summed for a raw score, which was then converted into a T-Score based on the Short Form Conversion Table. Higher scores reflect a greater degree of anxiety or depression.

### 2.4. Analysis Plan

In our analysis, we assessed the change in HRQoL, and PROMIS score as an absolute difference from baseline assessment to the closest follow-up. A positive change indicates an increase (improvement) and negative change indicates a decrease from baseline. We implemented our analysis by grouping patients based on the source of the survey, either subject or parent. This was intentional as for most of the younger patients, and the parents’ survey answers were considered. The study was not powered to detect statistical significance.

## 3. Results

### 3.1. Descriptive Statistics

A total of 26 participants were enrolled in the study, composed of 16 unique patients and 10 caregivers. Detailed demographic details about the cohort are presented in Table 1. There were 10 individual subjects with baseline assessment and at least one follow-up assessment. There were 5 parent assessments (ages 8 and 18) and 7 BTHS subjects’ own assessments (ages 6–38 years). In addition, both parent and subject assessments were available for two subjects (ages 16 and 17). Table 2 depicts the subject’s age at the time of enrollment and the earliest follow-up available.

### 3.2. Pearson Correlations

Correlations among measures can be found in Supplemental Table S1. HRQoL physical and emotional scores at Baseline (T1) were significantly correlated with all other aspects of T1 QoL and negatively correlated with anxiety (*p*’s < 0.001). In addition, social score at T1 was negatively correlated with T1 depression (*p* < 0.01). At the earliest follow-up (T2), the only significant correlations that remained were T2 physical and school scores (*p* < 0.01), as well as T2 anxiety, T2 depression and T2 school scores (*p*’s < 0.05). Scores in the same category were not correlated across T1 and T2, except for physical score and social score (*p*’s < 0.01).

### 3.3. Score Change over Time

**Figure 1:**
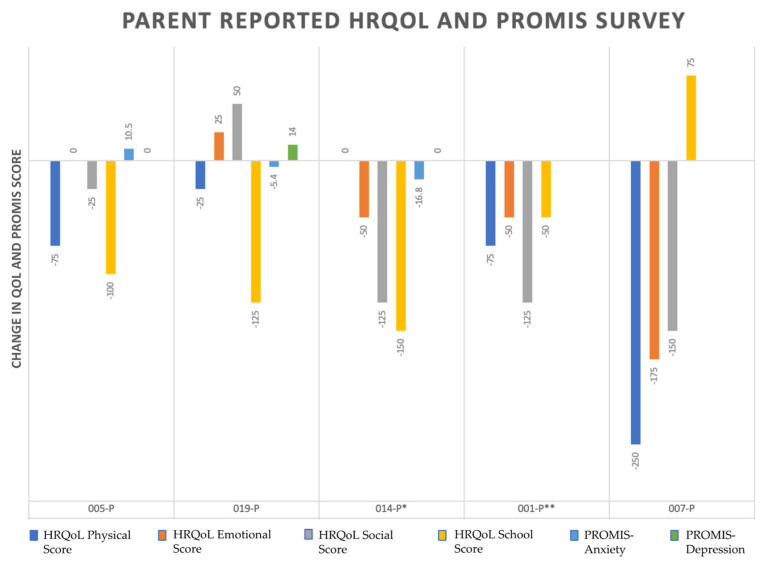
Change Over Time in Caregiver-reported HRQoL and PROMIS scores. P—parent, * and ** parent corresponding to paired subject.

**Figure 2:**
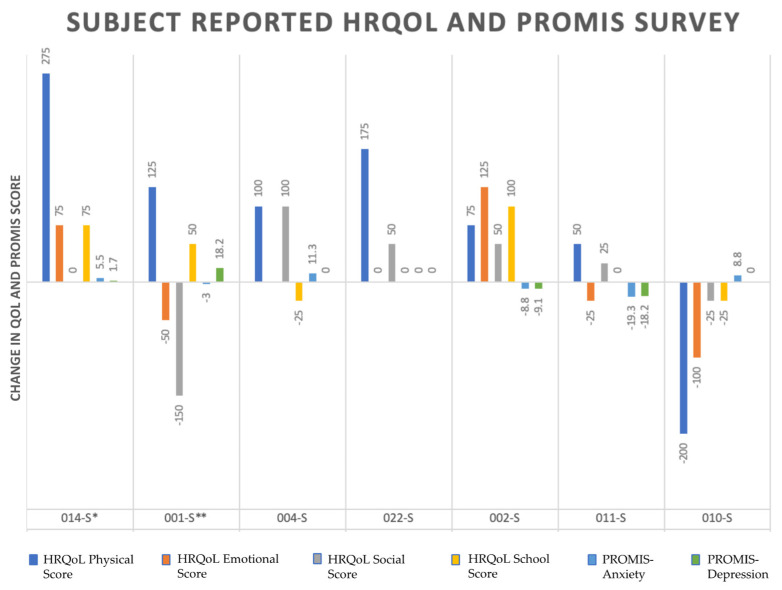
Change Over Time in Self-reported HRQoL and PROMIS scores. S—subject, * and ** subject corresponding to paired parent.

Figure 1 and Figure 2 summarizes the change in HRQoL scores (physical, emotional, social, school) and anxiety and depression scores as ‘follow-up score–baseline score’.

Based on the reported scores from 7 subjects at age ranging from 16 to 38, the physical score improved in 6 of 7 cases. Based on parent-reported results, the same score decreased in 4 of 5 cases between ages 8 and 18. There were significant discrepancies in parent and subject-reported results for two subjects (ID 14 and 1) with both parent and subject-based results. For case 001 in 6-month follow-up, the subject reported +125 change in physical score. In contrast, the parents reported −75 change. For the same score and follow-up time for case 014, the subject reported +275 change while the parent-reported results indicate no change from baseline (0).

The social score was primarily decreased based on parent-reported results (4 of 5), while it was mainly improved based on subject-reported results (4 of 7). Based on parent-reported results, school score was decreased in 4 of 5 cases. However, the school score was improved in 3 of 7 cases based on subject-reported results, which acknowledged that most of these cases might not be at school age. Interestingly, for cases 001 and 014 with available parent- and subject-based results, the school performance was improved according to subjects while it decreased according to parents.

Anxiety scores were available for 9 out of 10 subjects. Four patients had worse scores from the baseline. For the patients for which we had both parent and subject responses available (case 014), parents reported worsening anxiety while the subject reported improvement from the baseline at the same follow-up interval. Depression scores showed worsening in only 2 out of 9 subjects. Five subjects reported no change from the baseline. Case 011 showed the most worsening anxiety and depression from the baseline compared to other subjects.

## 4. Discussion

Comprehensive care should be the goal of providers managing patients with chronic diseases such as BTHS. Managing recognized pathophysiologic conditions such as neutropenia/infection, cardiomyopathy, and heart failure are essential and continue to be an area of ongoing clinical investigation. However, very little attention has been given to the other BTHS comorbidities that have been shown to impact other chronic diseases significantly.

Thoughtful BTHS care in the current era must include longitudinal assessment of the quality of life and screening for affective symptoms and psychiatric diagnoses. The identification of abnormalities in these domains affords providers another avenue for screening. These screening efforts can lead to interventions that drastically change overall health status and impact disease course. For example, previous experience in the largest outpatient cardiomyopathy cohort in the United States has identified decreased quality of life scores in ~50% of the population and abnormal anxiety and depression scales in ~30%. Furthermore, interventions such as counseling and education have improved these scores over time. This same opportunity exists in the BTHS community and should be pursued to deliver the highest level of comprehensive care possible.

In this study, we provide preliminary evidence for a pattern of discordance between patient and caregiver-reported outcomes. Most subjects reported improvement in the HRQoL measures, with parents reporting declines. In the age group of 16–17 years, for which we have both patient and parent-reported data, we document patients reporting psychosocial improvement in contrast to parental responses for the same patients. Although this analysis was based on two caregiver-patient dyads, the data suggests a pattern of differences in perceived quality of life between the patients and parents. The differences that we saw could also be dependent on the patient’s age, with older patients who are better able to comprehend their quality of life with the disease course reporting favorable and improved psychosocial measures. Our data also indicates that the disease course and its impact on HRQoL can be variable and may be informed by other factors not captured by our survey.

The association between mood disorders and medication non-adherence has been studied previously. DiMatteo and colleagues found that compared to non-depressed patients, depressed patients were three times more likely to be non-adherent to medical recommendations. While this was not directly measured in our study, the data did not show a direct correlation between the physical quality of life scores and changes in anxiety and depression scores [27].

There are obvious limitations to our study. Our study suffered from a small sample size. Nevertheless, given the exceptionally low prevalence of BTHS, we feel this is a good snapshot of a scarce population. Alongside the small sample size and a recognized lack of uniformity and follow-up, the intervals between baseline and follow-up evaluations were inconsistent. This is partly secondary to an international population with limited access to care. This challenged us to contact these patients and their caregivers for follow-up survey inquiries. Given that our results show a trend toward discordance between the available caregiver versus patient-reported outcomes, additional studies with a larger sample size are warranted to replicate our findings. This discordance between the patient and parent proxy responses has been reported in children with heart disease [28]. The study also suffered from a limited number of patients and families providing follow-up information from baseline interrogation. Future studies in the BTHS population should target a more reliable mechanism for patient enrollment and follow-up evaluation.

This report is the first to characterize QoL and PROMIS scores over time with reports from patients and caregivers. Importantly, we found heterogeneity both amongst patients and when comparing patients to caregivers. This stresses the necessity to assess the BTHS population sequentially with surveys such as those leveraged in our study to define better changes in quality of life and potential indicators of anxiety and depression. Our study data shows that scores reported by parents differed from patient-reported data. Almost uniformly, the caregiver’s perception of the changes was more negative the than the patient’s own perception, as indicated by the scores. While the small sample size limits our ability to draw definitive conclusions from the data, it offers compelling evidence to broaden the current clinical care delivery in this complex and growing cohort. Considering BTHS is a rare disorder, even this limited data provides valuable insights into the psychosocial and mental health component associated with the natural history of this disease.

## 5. Conclusions

BTHS is a rare genetic disorder with a wide range of phenotypic variations. Our study provides valuable insights into the impacts of psychosocial and mental health aspects of BTHS. Quality of life changed significantly over time in various domains, both in terms of improvements and declines. Many subjects in our study experienced significant changes in scores related to anxiety and depression. These results inform us of the clinical pathway available for appropriate and necessary care of the BTHS population.

## Figures and Tables

**Table 1:** Participant Demographics.

	*n* (%)	Mean (SD; Range)
**Sex**		
Male	16 (100%)	
Female	0 (0%)	
**Age**		21.25 (12.66; 7–51)
≤14	5 (31.25%)	
15–19	5 (31.25%)	
20–29	2 (12.5%)	
30–39	3 (18.75%)	
40–49	0 (0%)	
50+	1 (6.25%)	
**Follow-up Period**		
Enrollment/Baseline	26	
6 months	7	
12 months	7	
18 months	3	
24 months	2	
30 months	3	
36 months	8	
**HRQoL (Baseline caregiver-report)**		
Physical Score	8	365.63 (262.18; 50–775)
Emotional Score	8	356.25 (101.55; 225–500)
Social Score	8	306.25 (117.83; 175–500)
School Score	8	315.63 (195.37; 200–500)
**HRQoL (Baseline self-report)**		
Physical Score	14	426.79 (210.19; 75–775)
Emotional Score	13	371.15 (87.11; 225–500)
Social Score	14	346.43 (97.01; 200–500)
School Score	14	316.07 (115.86; 150–500)
**PROMIS (Baseline caregiver-report)**		
Anxiety	5	45.90 (15.60; 34.60–65.60)
Depression	5	36.20 (0; 36.20–36.20)
**PROMIS (Baseline self-report)**		
Anxiety	14	46.26 (8.60; 33.50–59.40)
Depression	14	43.11 (7.80; 35.20–59.70)

**Table 2:** Participant Enrollment Age and Follow-up Time.

	ID	Subject Age at Enrolment (years)	Earliest Follow Up (months)
Parent	005-p	8	30
	019-p	8	24
	014-p *	16	6
	001-p **	17	6
	007-p	18	12
Subjects	014-s *	16	6
	001-s **	17	6
	004-s	29	6
	022-S	29	30
	002-s	30	6
	011-s	34	12
	010-s	38	6

* and ** parent corresponding to paired subject.

## Data Availability

Not applicable.

## Supplementary Materials

The following supporting information can be downloaded at: https://www.mdpi.com/article/10.3390/jcdd9120448/s1, Table S1: Pearson Correlation Coefficients among Key Measures.
