# Supplementary material for: Barth Syndrome: Psychosocial Impact and Quality of Life Assessment
Source: J Cardiovasc Dev Dis. 2022 Dec 9;9(12):448. doi: 10.3390/jcdd9120448 (PMC9784194; doi:10.3390/jcdd9120448)
Supplement: Supplementary file 1 [file jcdd-09-00448-s001.zip › jcdd-1991222-supplementary.pdf]

Supplemental Table

Table S1. Pearson Correlation Coefficients among Key Measures

	**1**	**2**	**3**	**4**	**5**	**6**	**7**	**8**	**9**	**10**	**11**	**12**	**13**
**1.Age**	-												
**2.T1 Physical**	.22	-											
**3.T1 Emotional**	.20	.84**	-										
**4.T1 Social**	.28	.58**	.61**	-									
**5.T1 School**	.10	.71**	.67**	.60**	-								
**6.T1 Anxiety**	.08	-.75**	-.69**	-.36	-.41	-							
**7.T1 Depression**	.20	-.40	-.38	-.52**	-.41	.35	-						
**8.T2 Physical**	.51	.75**	.50	.50	.51	-.68*	.09	-					
**9.T2 Emotional**	.18	.50	.60	.27	.41	-.72*	-.10	.65*	-				
**10.T2 Social**	.67*	.51	.41	.74**	.43	-.37	.08	.71**	.51	-			
**11.T2 School**	.63*	.82**	.57	.35	.45	-.66*	.02	.65*	.58*	.54	-		
**12.T2 Anxiety**	-.32	-.44	-.50	-.30	-.55	.48	.24	-.33	-.71*	-.33	-.60	-	
**13.T2 Depression**	-.23	-.40	-.51	-.26	-.64*	.48	.35	-.13	-.58	-.20	-.42	.89**	-

** Denotes significance at $p < .05$*
*** Denotes significance at $p < .01$*
T1 refers to score at enrollment
T2 refers to score earliest follow-up
